# Half of rifampicin-resistant *Mycobacterium tuberculosis* complex isolated from tuberculosis patients in Sub-Saharan Africa have concomitant resistance to pyrazinamide

**DOI:** 10.1371/journal.pone.0187211

**Published:** 2017-10-31

**Authors:** Jean Claude Semuto Ngabonziza, Awa Ba Diallo, Elisa Tagliani, Bassirou Diarra, Abalo Essosimna Kadanga, Antieme Combo George Togo, Aliou Thiam, Willem Bram de Rijk, Riccardo Alagna, Sabine Houeto, Fatoumata Ba, Anoumou Yaotsè Dagnra, Emil Ivan, Dissou Affolabi, Valérie Schwoebel, Arnaud Trebucq, Bouke Catherine de Jong, Leen Rigouts, Géraldine Daneau, the “Union short MDR-TB regimen study group”

**Affiliations:** 1 National Reference Laboratory Division, Biomedical Services Department, Rwanda Biomedical Centre, Kigali, Rwanda; 2 Mycobacteriology Unit, Department of Biomedical Sciences, Institute of Tropical Medicine, Antwerp, Belgium; 3 Mycobacteriology Unit, Bacteriology- Virology Laboratory, CHNU Aristide le Dantec, Dakar, Senegal; 4 Emerging Bacterial Pathogens Unit, Division of Immunology, Transplantation and Infectious Diseases, IRCCS San Raffaele Scientific Institute, Milan, Italy; 5 SEREFO/UCRC Program, University of Sciences, Techniques and Technologies of Bamako, Bamako, Mali; 6 Laboratoire National de Référence de Mycobactéries, Lome, Togo; 7 Laboratoire de Référence des Mycobactéries, Cotonou, Benin; 8 Laboratoire de Reference des Mycobactéries, Dakar, Senegal; 9 International Union Against Tuberculosis and Lung Disease, Paris, France; 10 Department of Biomedical Sciences, Antwerp University, Antwerp, Belgium; 11 Biomedical section, Haute Ecole Francisco Ferrer, Brussels, Belgium; Institut de Pharmacologie et de Biologie Structurale, FRANCE

## Abstract

**Background:**

Besides inclusion in 1^st^ line regimens against tuberculosis (TB), pyrazinamide (PZA) is used in 2^nd^ line anti-TB regimens, including in the short regimen for multidrug-resistant TB (MDR-TB) patients. Guidelines and expert opinions are contradictory about inclusion of PZA in case of resistance. Moreover, drug susceptibility testing (DST) for PZA is not often applied in routine testing, and the prevalence of resistance is unknown in several regions, including in most African countries.

**Methods:**

Six hundred and twenty-three culture isolates from rifampicin-resistant (RR) patients were collected in twelve Sub-Saharan African countries. Among those isolates, 71% were from patients included in the study on the Union short-course regimen for MDR-TB in Benin, Burkina Faso, Burundi, Cameroon, Central Africa Republic, the Democratic Republic of the Congo, Ivory Coast, Niger, and Rwanda PZA resistance, and the rest (29%) were consecutive isolates systematically stored from 2014–2015 in Mali, Rwanda, Senegal, and Togo. Besides national guidelines, the isolates were tested for PZA resistance through *pncA* gene sequencing.

**Results:**

Over half of these RR-TB isolates (54%) showed a mutation in the *pncA* gene, with a significant heterogeneity between countries. Isolates with fluoroquinolone resistance (but not with injectable resistance or XDR) were more likely to have concurrent PZA resistance. The pattern of mutations in the *pncA* gene was quite diverse, although some isolates with an identical pattern of mutations in *pncA* and other drug-related genes were isolated from the same reference center, suggesting possible transmission of these strains.

**Conclusion:**

Similar to findings in other regions, more than half of the patients having RR-TB in West and Central Africa present concomitant resistance to PZA. Further investigations are needed to understand the relation between resistance to PZA and resistance to fluoroquinolones, and whether continued use of PZA in the face of PZA resistance provides clinical benefit to the patients.

## Introduction

Although efficient chemotherapy has been available since 1965, tuberculosis (TB) remains one of the most threatening infectious diseases [1]. TB control and management is challenged by the ongoing spread of drug-resistant TB isolates. Each year, an estimated 480,000 new cases of multi-drug resistant (MDR) TB occur worldwide, with more than 10% of these cases being extensively drug-resistant (XDR) TB [1]. Since May 2016, the World Health Organization (WHO) recommends a 9-month regimen developed by the International Union against Tuberculosis and Lung disease after successful experiences in Bangladesh [2,3]. The short regimen consists of a minimum of 4 months of kanamycin (KM), clofazimine (CFZ), moxifloxacin (MFX), ethambutol (EMB), high-dose isoniazid (INH), pyrazinamide (PZA) and prothionamide (PTH) followed by 5 months CFZ, MFX, EMB and PZA [4].

PZA is an indispensable drug for the treatment of both drug susceptible and MDR-TB cases. This sterilizing drug plays a key role in reducing TB relapse rates and shortening the standard course of 1^st^ line TB treatment from 9–12 months to 6 months [5]. In addition, PZA is the only 1^st^ line anti-TB drug most likely to be maintained in all new regimens, including those aiming at reducing the treatment duration for rifampicin susceptible-, MDR- and XDR-TB [6]. However, several studies have shown a clear association between resistance to PZA and to rifampicin (RMP), with the proportion of PZA resistance among RMP resistant (RR) greater than 40% in different settings[7–9].

Although resistance to PZA in RR-TB has been associated with poor treatment outcome in some settings, the drug susceptibility test (DST) is rarely done [10,11]. Phenotypic detection of PZA resistance is difficult and often unreliable, as the drug is active only at pH 5.5 acidity, which affects *in vitro* growth of *Mycobacterium tuberculosis*, causing both false-susceptible and false-resistant results [12,13]. PZA is a pro-drug that is converted to pyrazinoic acid by the pyrazinamidase/nicotinamidase enzyme encoded by the *pncA* gene of *M*. *tuberculosis*. A mutation in *pncA* can confer PZA resistance by decreasing the enzyme activity, either by impeding the function (mutation in the coding sequence) or by reducing protein production (mutation in the promoter region) [14]. A variety of different *pncA* mutations are found in 72–97% of phenotypically resistant clinical isolates [15]. Studies have reported that, within an MDR-TB population *pncA* sequencing has a good diagnostic accuracy of 89.5–98.8% for PZA resistance[16–19]. Hence, *pncA* gene sequencing has been proposed as a quick and reproducible method for PZA resistance detection from either microscopy-positive clinical samples or bacterial isolates [9,11]. Sequencing of *pncA* is an acceptable and reliable approach to detect PZA resistance particularly when considering that conventional phenotypic PZA susceptibility testing is difficult and prone to false resistance [20,21]. Given the increased availability of sequencing technology, genotypic-based testing for PZA resistance will thus likely become the method of choice for PZA DST.

Unfortunately, PZA resistance has not been widely investigated in most African countries, which bear most of the TB and HIV burden globally. Knowing the prevalence of PZA resistance and the variety of *pncA* mutations would help to inform an optimized diagnostic and treatment strategy in Africa.

In this multi-center study, we analyzed the *pncA* sequences of the RR isolates from 12 Sub-Saharan African countries. Among the RR isolates considered for this study, 448 (72%) were from the MDR-TB clinical study coordinated by the Union conducted from 2012 to 2015 in nine countries, which had *pncA* sequenced by the respective TB Supranational reference laboratories (SRL)[3], and 175 (28%) isolates were collected from consecutive RR patients from 2014 to 2015 in four countries as part of routine drug resistance surveillance and/or for research purposes, with sequencing analysis in the own country

## Material and methods

### Study population

**Fig 1:**
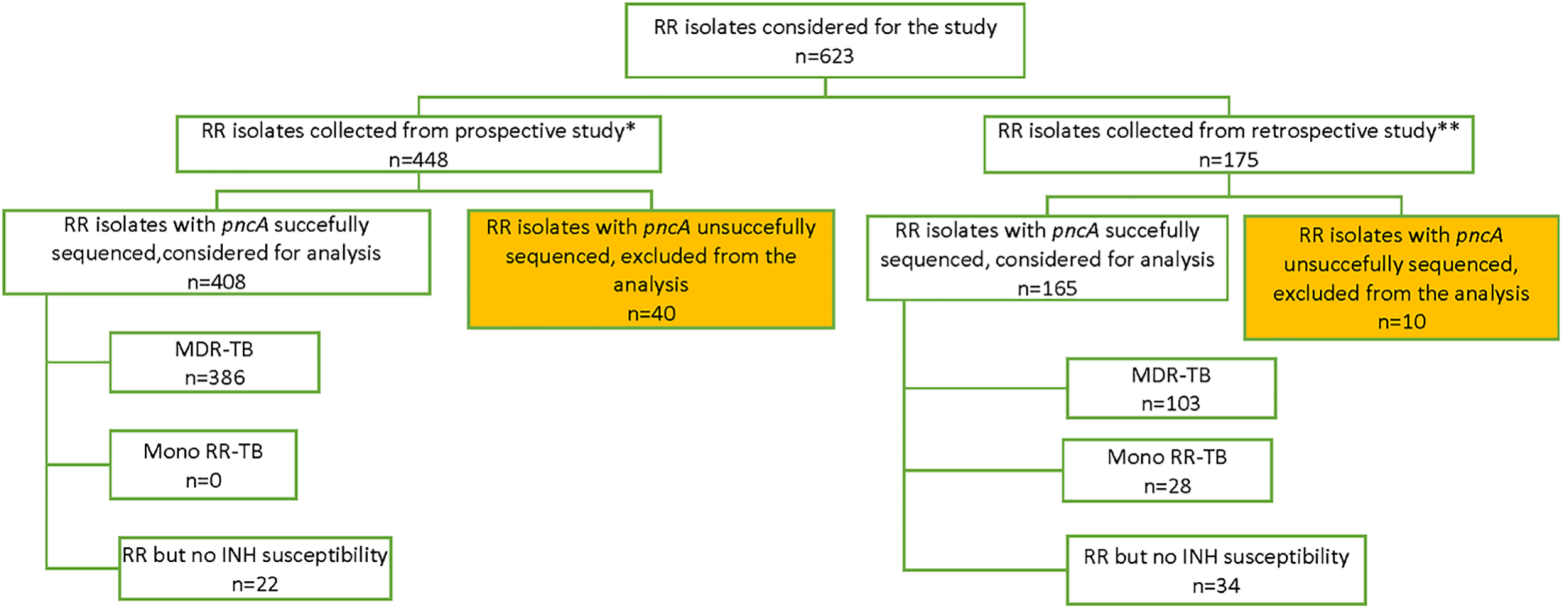
Flow chart of the samples included in our study. INH: isoniazid. RR: Rifampicin Resistance. MDR-TB: Multidrug resistant tuberculosis (*M*. *tuberculosis* resistant to both rifampicin and isoniazid). *Prospective study: An MDR-TB clinical study coordinated by the Union from 2012 to 2015 in nine African countries. **Retrospective study: consecutive RR-TB samples stored from 2014 to 2015 as part of routine drug resistance surveillance and/or for research purposes.

Samples were collected in twelve countries in Sub-Saharan Africa, through two different studies: one prospective on patients starting the short-course regimen for MDR-TB [3], and one retrospective on RR isolates stored from routine surveillance in 2014 and 2015 (Fig 1). For each country, demographic data collected included age, sex, HIV status, and history of TB treatment.

Studies were approved by the following ethical committees: Comité d’éthique pour la recherche en santé (délibération no 2013-09-013, Ouagadougou, Burkina Faso), Comité national d’éthique pour la recherche en santé (avis éthique favorable no 024 du 27 Sept 2012, Cotonou, Benin), Comité national d’éthique pour la protection des êtres humains participants à la recherché biomédicale et comportementale (décision du comité national d’éthique en date du 18/02/2012, Bujumbura, Burundi), Comité scientifique chargé de la validation des protocoles et résultats d’étude (no 24/UB/FACSS/CSCVPRE/12, Bangui, République Centrafricaine), Comité National d’éthique de la recherche pour la santé humaine (2013/01/no 001/CI/CNERSH/SP, Yaoundé, Cameroun), Comité d’éthique (no d’approbation: ESP/CE/061/2012, Kinshasa, République Démocratique du Congo), Comité consultatif national d’éthique (délibération no 015/2012/CCNE, Niamey, Niger), Comité national d’éthique et de la recherche (no 5263/MSLS/CNER-dkn, Abidjan, Cote d’Ivoire), National Ethics Committee (IRB 00001497 of IORG0001100, no 464/RNEC/2013, Kigali, Rwanda) and Ethical Advisory Group of the Internal Union Against Tuberculosis and Lung Disease (EAG no: 50/12, Paris France). All patients included signed an informed consent form. The retrospective study was approved by the Institutional Review Board at the Institute of Tropical Medicine (IRB/AB/ac/094 Ref: 1100/16, Antwerp, Belgium) and National Ethics Committee (IRB 00001497 of IORG0001100, no 674/RNEC/2016, Kigali, Rwanda). As these surplus samples from routine diagnostics were used retrospectively and anonymously, no informed consent was required. In Mali, patients had been recruited in specific ethically approved research protocols, with varying inclusion/exclusion criteria.

### Sampling coverage

RR -TB patients identified over the course of the routine drug resistance surveillance conducted in Rwanda, Senegal, and Togo were representative of the whole country. For Rwanda and Togo, they included both new TB cases and patients with a previous history of TB (retreatment cases), while for Senegal, only retreatment patients were sampled.

Samples from the Center for TB and AIDS Research in Mali within the University Clinical Research Center (SEREFO/UCRC) were not representative of the global MDR-TB population in Mali.

For the Union short course study, consecutive patients with RR were systematically invited to participate. Children, pregnant women, patients having received second-line TB drugs for more than one month, patients with medical and/or social contra-indication, or refusing to give informed consent, were excluded. Samples were sent to the respective SRLs in Cotonou, Milan or Antwerp for laboratory testing not available on site.

### Resistance testing

Sputum samples were cultured in the respective national reference laboratories using Mycobacterial growth indicator tube (MGIT, Middlebrook 7H9) (BD Biosciences, Erembodegem, Belgium) and/or in-house prepared Löwenstein Jensen slants. Positive cultures were identified for *M*. *tuberculosis complex* based on MPT64 (BD Biosciences, or SD Bioline, Gyeonggi-do, Republic of Korea), or Capilia^™^ TB-Neo Assay, Tauns Laboratories, Japan. As confirmed RR-TB patients were enrolled, they all had 1^st^ line DST done as part of screening, according to national regulations, using GeneXpert (Cepheid, Sunnyvale, CA), MGIT, LJ, and/or MTBDR*plus* (Hain Lifescience, Nehren, Germany), and most isolates were tested for 2^nd^ line DST using LJ, MTBDRsl (Hain Lifescience, Nehren, Germany), and/or target gene sequencing. The critical concentrations used to determine the phenotypic susceptibility to the 1^st^ and 2^nd^ line drugs tested are captured in the S2 File. HIV status was also determined per the national HIV testing algorithm. Spoligotyping was performed on samples from the same country with the same *pncA* mutation, based on previously described procedures [22].

Sanger sequencing was performed on DNA from cultured isolates (Primer list in S2). DNA from the Union study isolates was amplified at respective SRLs (Institute of Tropical Medicine, Antwerp, Belgium, and San Raffaele Scientific Institute, Milan, Italy), and sequencing outsourced respectively to BaseClear (Leiden, The Netherlands) and Eurofins Genomics (Ebersberg, Germany), with analysis at SRL by comparing obtained sequences to the reference *pncA* sequence from *M*. *tuberculosis* H37Rv (GenBank accession no. NC_000962.3) using BioEdit software (Ibis Biosciences, Abbott company, Carlsbad, CA), or CLC Sequence Viewer (CLCbio, Qiagen, Redwood City, CA), including at least 41 nucleotides from the promoter region. DNA from retrospective isolates was amplified in in-country reference laboratories, and sequencing was outsourced to Macrogen Europe (Amsterdam, the Netherlands). After organizing several trainings on generation and analysis of sequences, the *pncA* sequences were analyzed at the in-country reference laboratories using MEGA6 [23]. The full procedures are available in S2 File.

Samples received in Milan were also analyzed by whole genome sequencing (WGS). For WGS, genomic DNA was extracted from cultured isolates using the QIAamp DNA Mini kit (Qiagen, Hilden Germany) according to manufacturer’s instructions. Prior to submission for WGS, DNA was quality assessed and quantified using the Qubit dsDNA BR assay (Life Technologies, Paisley, UK). Paired-end libraries of 100 bp read length were prepared using the Nextera XT DNA Sample Preparation kit (Illumina Inc., San Diego, CA, USA) and sequenced on an Illumina HiSeq 2500 platform according to the manufacturer's instructions.

Downstream analysis was performed using available online analysis pipeline[24] including quality control checks. A mean read coverage depth of at least >30x. was considered acceptable. The reads obtained were then aligned to *M*. *tuberculosis* H37Rv reference genome, and total variant calling was performed with the pipeline of the PhyResSE web-tool. Isolates showing a coverage of at least 20× were considered for SNP analysis.

Interpretation of PZA resistance based on *pncA* mutations was based on published literature (S3 File) [5,9,15,25–32]. Silent mutations were considered as not associated with resistance, in addition to several mutations known not to confer phenotypic resistance [5,9,15,25–32]. Non-silent mutations not previously reported, or reported with discordant associations with phenotypic resistance, were considered associated with resistance in the remainder of the analysis.

### Data management and analysis

Data from the Union study were merged with retrospective data into a single Excel sheet database. All samples with poor quality *pncA* sequences (non-analyzable) were excluded from analysis. Descriptive statistics and calculation of different proportions were done for aggregated data in different categories and sub-categories. The Pearson’s chi-square and Fisher's exact tests were used to test for associations of PZA resistance with potential risk factors. Data analysis was performed using STATA version 14.2 software (College Station, TX: STATA Corp.), and a p-value <0.05 was considered significant.

## Results

### Study population and sampling coverage

Among 623 isolates considered for this study, 50 (8%) were excluded from analysis due to non-analyzable *pncA* sequences, due to poor quality of the sequence. Of the 573 (92%) included for analysis, 408 (71%) originated from the Union study of whom 342 (84%) were isolated from previously treated TB patients. For the remaining 165 isolates collected from routine surveillance of drug resistance, the treatment history of the patient was unknown for 56 (34%), and 72 of 109 (66%) isolates with documented treatment history were collected from new TB patients, with the majority (42 of 72, 58%) of new TB patients originating from Rwanda.

Most samples were tested for INH, with majority of them being resistant (proven MDR-TB) (Fig 1). However, as some were INH-sensitive (proven RR-TB), and some samples had no data for INH, we considered our sampling as RR-TB rather than MDR-TB.

Considering the resistance profile to 2nd line anti-TB drugs, 8 (1.4%) isolates were XDR, and 36 (6.3%) were pre-XDR with resistance to either fluoroquinolones (FQs) (n = 28) or second line injectable (SLI) (n = 8). However, the FQ and SLI susceptibility was not known for 141 (25%) isolates (Table 1). There was neither a difference in successfully versus failed sequenced samples, nor in the proportion of new versus previously treated patients, nor between the Union study versus the retrospective collection (p > 0.05).

**Table 1:** Sampling fraction and additional resistance among RR-TB patients in the study.

Country	n^1^^,^^2^ (%) stored	n^3^ (%) included	n (%) new cases	n (%) pre-XDR^4^	n (%) XDR^4^
**Benin**	15 (52)	14 (93)	0 (0)	NA^5^	NA
**Burkina Faso**	29 (85)	5 (17)	0 (0)	0 (0)	0 (0)
**Burundi**	34 (56)	32 (94)	0 (0)	0 (0)	0 (0)
**Cameroon**	145 (82)	133 (92)	9 (6.7)	2 (1.5)	0 (0)
**CAR**	37 (82)	35 (95)	0 (0)	0 (0)	0 (0)
**DRC**	102 (34)	95 (93)	16 (17)	16 (17)	2 (2.1)
**Ivory Coast**	162 (62)	31 (19)	0 (0)	14 (45)	4 (13)
**Mali**	NA	21	2 (9)	NA^6^	2 (9.5)
**Niger**	59 (95)	48 (81)	1(2)	4 (8)	0 (0)
**Rwanda**	108 (47)	101 (94)	9 (60)	0 (0)	0 (0)
**Senegal**	NA	39	1 (2.3)	NA^5^	NA
**Togo**	20 (95)	19 (95)	1 (5.3)	NA^5^	NA
**Total**	**711**	**573 (81)**	**72 (13)**	**36 (6.3)**	**8 (1.4)**

Number (n) and percentage (%) for samples at each study step.

DRC: The Democratic Republic of the Congo. CAR: Central African Republic. Pre-XDR: resistant to either fluoroquinolone or second line injectable (SLI). XDR: resistant to both fluoroquinolone and SLI. NR: not representative. NA: not applicable.

^1^ The number of samples received at the Supranational Reference Laboratories as proportion of the number of patients recruited.

^2^Number of samples stored versus numbers diagnosed per the WHO report for the same period for the whole country.

^3^Number of isolates with analyzable sequences as proportion of total samples stored.

^4^The proportion of pre-XDR or XDR in these columns corresponds to the ratio of pre-XDR or XDR to the total isolates included in this analysis, it therefore does not reflect the general prevalence of pre-XDR or XDR TB in the respective countries.

^5^FQ was not known.

^6^FQ was not known for all isolates (partial).

The male to female sex ratio in our sequenced population was 1.8, and the median age was 33 years (Interquartile range 26–42). The HIV status was known for 521 (91%) patients, with 111 (21.3%) patients being HIV-TB co-infected. The rate of HIV co-infection among participants was different between countries (p < 0.001) (Table 2).

**Table 2:** Demography of RR-TB patients included in *pncA* analysis.

Country	n	Median age (range)	n male sex (%)	n HIV-infected (%)
**Benin**	14	32 (21–45)	11 (79)	3 (20)
**Burkina Faso**	5	44 (29–55)	5 (100)	0
**Burundi**	32	28 (18–44)	16 (55)	6 (21)
**Cameroon**	133	32 (18–60)	76 (61)	35 (28)
**DRC**	95	31 (18–75)	51 (61)	13 (16)
**CAR**	35	32 (18–49)	19 (61)	5 (16)
**Ivory Coast**	31	33 (22–63)	19 (66)	2 (7)
**Mali**	21	32 (19–64)	13 (62)	1 (5)
**Niger**	48	36 (18–70)	33 (70)	6 (13)
**Rwanda**	101	36 (14–80)	68 (68)	36 (42)
**Senegal**	39	29 (8–65)	27 (75)	0
**Togo**	19	34 (20–60)	10 (53)	4 (21)
**Total**	573	33 (8–80)	348 (65)	111 (21)

DRC: The Democratic Republic of the Congo. CAR: Central African Republic.

### Resistance testing

Among the 573 isolates successfully sequenced, 311 (54%) presented mutations in the *pncA* gene, of which 248 (80%) were previously reported in the literature [5,9,15,25–32]. Of the reported mutations, 12 (4.8%) were clearly defined as not associated with PZA resistance. Excluding those “non-resistance conferring” mutations for the resistance prevalence estimates, 299 (52%) isolates were PZA resistant. The prevalence of PZA resistance was similar in the Union study and among the retrospective isolates (p = 0.347), yet significantly differed between countries (p<0.001, Table 3).

**Table 3:** Number of sequences presenting a mutation in *pncA*.

Country	n with *pncA* mutation (% of total, CI)	n previously reported in literature	n associated	n with unclear association	n not associated	n new	Total n mutations considered associated (%, CI)
**Benin**	5 (33, 12–62)	4	3	1	0	1	5 (36, 13–65)
**Burkina Faso**	4 (80, 28–99)	3	3	0	0	1	4 (80, 28–99)
**Burundi**	22 (69, 50–84)	14	12	0	2	8	20 (63, 44–79)
**Cameroon**	66 (50, 41–58)	53	46	6	1	13	65 (49, 40-58-55)
**CAR**	10 (29, 15–46)	8	5	2	1	2	9 (26, 12–43)
**DRC**	70 (74, 64–82)	54	50	2	2	16	68 (72, 61–80)
**Ivory Coast**	17 (55, 36–72)	13	7	5	1	4	16 (52, 34–69)
**Mali**	13 (62, 37–82)	12	12	0	1	0	12 (57, 34–78)
**Niger**	19 (40, 26–55)	12	10	1	1	7	18 (38, 24–53)
**Rwanda**	68 (67, 57–76)	62	61	0	1	6	67 (66, 56–75)
**Senegal**	13 (33, 19–50)	12	7	3	2	1	11 (28, 15–45)
**Togo**	4 (21, 6–45)	0	0	0	0	4	4 (21, 6–46)
**Total**	**311 (54, 50–58)**	**247**	**216**	**20**	**12**	**64**	**299 (52, 48–56)**

Number (n) of samples presenting a mutation, and categorization based on the literature (S3 File). Percentage (%, and 95% confidence interval) of all samples with a successful *pncA* sequence.

Unclear association: pncA mutation reported as both associated and not associated with resistance in literature

New: pncA mutation not previously reported in literature

Mutation considered associated: all mutations except those not associated.

Among these RR patients, the prevalence of PZA resistance was similar in new versus previously treated patients (p = 0.167), and in HIV-infected versus uninfected patients (p = 0.946). However, the prevalence was higher among isolates showing resistance to FQ (p < 0.001) but not for those resistant to SLI (p = 0.174) or resistant to both FQ and SLI (p = 0.399) (Table 4).

**Table 4:** Number of samples with PZA resistance by category of patients.

Country	n (%, CI) in new cases	n (%, CI) in retreatment	n (%, CI) in pre-XDR FQ-R	n (%, CI) in pre-XDR SLI-R	n (%, CI) in XDR	n (%, CI) in HIV+
**Benin**	-	5 (36, 13–65)	NA	NA	NA	0
**Burkina Faso**	-	4 (80, 28–99)	0	0	0	0
**Burundi**	-	17 (59, 39–76)	0	0	0	1 (16, 0.4–64)
**Cameroon**	5 (56, 21–86)	58 (50, 40–59)	2 (100, 16–100)	0	0	15 (43, 26–61)
**CAR**	-	7 (23, 10–41)	0	0	0	2 (40, 5.3–83)
**DRC**	12 (75, 48–93)	46 (69, 56–79)	15 (94, 70–100)	4 (100, 40–100)	2 (100, 16–100)	9 (69, 39–91)
**Ivory Coast**	-	16 (55, 36–74)	8 (67, 35–90)	6 (60, 26–87)	2 (50, 6.7–93)	2 (100, 16–100)
**Mali**	-	12 (57, 34–78)		NA	1 (5, 0.1–24)	2 (100, 16–100)
**Niger**	-	18 (39, 25–55)	3 (75, 19–99)	0	0	1 (17, 0.4–64)
**Rwanda**	29 (69, 53–82)	24 (67, 49–81)	0	0	0	26 (72, 55–86)
**Senegal**	-	11 (31, 17–49)	NA	NA	NA	0
**Togo**	-	4 (22, 6.4–48)	NA	NA	NA	0
**Total**	46 (64, 52–75)	222 (49, 45–55)	28 (82, 65–93)	10 (71, 42–92)	5 (62, 24–91)	58 (52, 42–61)

Number (n) of samples presenting a mutation considered associated, and categorization by patient treatment history and resistance profile. Proportion (%, and 95% confidence interval) of total number of samples with a *pncA* sequence for the respective country.

Definitions of categories. New cases: patients never treated before or treated for less than one month. Retreatment: relapse or failure of previously treatment. FQ-R: isolate resistant to fluoroquinolones. SLI R: isolate resistant to second-line injectables. XDR: isolate resistant to both FQ and SLI. HIV+: isolates from HIV co-infected patients. NA: not applicable.

Some mutations were found in more than one patient from the same country (defined as the same NRL or research center). Considering also the resistance profile for other genes, we observed 40 clusters, with 2 to 14 samples in each cluster (Table 5). In five countries, more than 25% of the retreatment cases were included in different clusters. Also, two large cross-border clusters were identified in neighboring countries; a group of 21 isolates in Rwanda and Burundi, and a group of 13 isolates in Rwanda and DRC. Spoligotyping of 11/13 isolates included in the two larger national clusters showed that most isolates in each cluster were identical (one isolate had a different spoligotype in each group), the cluster in Burundi from the LAM11 family, and the one in Cameroon from *M*. *africanum* (L5).

**Table 5:** Number of clusters with identical *pncA* and others genes mutations.

Country	n groups	Range n/cluster	n (%, CI) of new cases	n (%, CI) of retreatment cases
**Benin**	0	0	0	0
**Burkina Faso**	1	2	0	2 (40, 5.3–85)
**Burundi**	3	2–8	0	12 (46, 27–67)
**Cameroon**	7	2–7	1 (2.7, 0.07–14)	22 (20, 13–28)
**CAR**	1	2	1 (50, 1.2–99)	1 (4.3, 0.1–22)
**DRC**	10	2–5	7 (26, 13–44)	18 (26, 16–38)
**Ivory Coast**	1	3	0	3 (14, 29–35)
**Mali**	3	2	0	7 (35, 15–59)
**Niger**	2	2	0	3 (6.6, 1.4–18)
**Rwanda**	9	2–14	20 (49, 33–65)	22 (56, 40–72)
**Senegal**	2	2	0	4 (10, 2.8–24)
**Togo**	1	3	0	3 (17, 3.6–41)
**Togo**	1	3	0	3 (17, 3.6–41)

Number (n) of clusters with samples from the same reference center presenting the same *pncA* mutation and the same resistance profile for other genes. Percentage (%, and 95% confidence interval) of new cases [or retreatment cases] part of a group versus a total of new cases [or retreatment cases] for the respective country.

## Discussion

Our findings show that, in West and Central Africa, about half of RR-TB patients face concurrent PZA resistance. With this work, we fill a geographically large knowledge gap, as data from this high burden TB region were previously very limited. While the average rate of PZA resistance was 52% (95% CI 48–56%) in RR-TB patients in this region, this rate varied from 21% in Togo to 80% in Burkina Faso, albeit with wide confidence intervals, and indeed differed significantly between countries (p<0.001). This level of PZA resistance, as well as the variations between countries, are in line with other regions [8,9]. Most patients had been previously treated (retreatment patients), although previous treatment was not associated with higher rates of PZA resistance. In Rwanda, the proportion of new TB patients was higher than in other settings, consistent with the national policy of testing all TB patients with GeneXpert. Similarly, HIV co-infection was not associated with PZA resistance. In contrast, PZA resistance was higher in isolates with concurrent resistance to FQ without SLI resistance (pre-XDR). Resistance to SLI had no significant influence, although numbers were low, yet this concurs with other studies [33,34].

A large proportion of the mutations observed had previously been reported, most reported to be associated with resistance, and a few as not associated with resistance. Still, 3.8% (95%CI, 2–6.6) were previously described mutations but with an unclear role in PZA resistance development. As the large majority of mutations reported in the literature are associated with resistance, and discordance may partially be due to non-reproducible phenotypic testing, we considered those as associated with resistance. Similarly, we considered all new (non-synonymous) mutations as associated with resistance. In addition, 31% of those new mutations cause a frame shift, which increases the likelihood that these confer resistance.

The exact impact of resistance to PZA on patient outcome is unclear. As the prevalence of PZA resistance is high in RR-TB patients, it is important to better understand whether any remaining activity warrants to continue PZA in the treatment regimen. A formal comparison in which patients are randomized to have PZA resistance results ‘ignored’ (and continue to receive PZA as part of their regimen), versus receiving a non-PZA regimen, would resolve this question yet may not be considered a priority for clinical study. In addition, laboratory testing should be strengthened if clinicians have to take PZA resistance results into account in selecting a regimen.

Also, the association with FQ resistance requires further study. FQs are a core class of drugs for RR-TB treatment and in the context of FQ resistance, additional resistance to other drugs may develop [30]. We hypothesize that PZA resistance is likely accumulated in RR/MDR TB patients who were not recognized as resistant and received multiple rounds of 1st line therapy containing PZA (including Category 2, with streptomycin). The association with FQ resistance suggests that PZA may protect against FQ resistance in PZA susceptible strains, although conversely, FQ resistance may also predispose to acquiring PZA resistance. With scaling up of earlier/ universal DST for rifampicin, already implemented in Rwanda, we expect that patients with RR/MDR would be diagnosed before PZA resistance develops, and that the prevalence of PZA resistance among RR/MDR would decline over time.

In addition to resistance testing itself, the high heterogeneity of *pncA* mutations also permits the use of *pncA* diversity to identify circulating MDR strains that are possibly actively transmitted, as the chance that the same mutation appears due to independent evolutionary events in different strains from the same region is low. In phylogenetic terms, *pncA* suffers less convergent evolution under drug pressure than other genes, such as *rpoB* and *katG*. Therefore, the *pncA* sequence has been proposed to be added to spoligotyping to identify clusters of transmission [35]. In our study, we observed several clusters of isolates presenting the same *pncA* mutation, and confirmed that the vast majority also shared the same spoligotype pattern. Those clusters likely reflect ongoing transmission, rather than reactivation (in new cases) or relapse (in retreatment cases), concurrent with reports that most MDR-TB is transmitted rather than acquired [36]. The overall low clustering rate in our study may reflect a low sampling fraction in each of the countries, or also be in line with the observations from den Hertog *et al*., suggesting that *pncA* mutations may confer a fitness loss preventing strains from wide dissemination [37]. Interestingly, one of the strains included in such a cluster was from lineage 5, known as *M*. *africanum* type 1, which is known to be less prone to tuberculosis disease progression[38]. Implementing *pncA* based DST for PZA may thus also assist the national program in recognizing whether predominant MDR-TB clusters reflect ongoing transmission in the communities, justifying targeted active case finding and other interventions.

Besides results collected for this analysis, the participating laboratories implemented the use of Sanger sequencing to identify mutations in TB-related genes. Staff from several NRL and research laboratories were indeed trained on sample preparation for (outsourced) sequencing and data analysis in 2013–2015, by the Institute of Tropical Medicine, Antwerp Belgium, in collaboration with the ITM TB network, WANETAM Plus and OFID Pasteur network. Even if sequencing facilities are expensive and scarcely available, we proved that a sequencing approach is possible for NRLs in Africa when outsourcing is used for the sequencing itself, and the staff may focus on the preparation of samples by PCR, and on the informatics’ analysis. As a resource for laboratory managers who would like to implement this approach in their own setting, our detailed procedures are added as S2 File. Furthermore, preliminary results at ITM indicate that the PCR can start directly from DNA extracted from smear-positive sputum samples.

We acknowledge the fact that *pncA* sequencing may not identify all existing resistant strains and, as a consequence, the overall levels of PZA resistance might be slightly underestimated. The analysis of additional genomic regions such as *rpsA*, *panD*, *hadC*, *fas1*, and the efflux pumps could increase the prediction of PZA resistance. However, as documented by several studies, we are convinced that further studies are needed to better understand the role of these targets and their real contribution to PZA resistance[39–42]. Methods including several genes in a single experiment based on next generation sequencing, like whole genome sequencing or multiple targeted sequencing, starting from culture or sputum, may be a more cost-effective method in the future for labs implementing the required skills, quality control, and material [43–46]. Whole genome sequencing will also allow the analysis of *panD* and other minor genes described in rare resistance to the active form of PZA [47–49].

In conclusion, this report on *pncA* mutations in West and Central Africa shows that the prevalence of mutations is similar to other regions in the world: half of RR-TB patients are also resistant to PZA. Further investigation will be needed to understand the relation between *pncA* mutations associated with PZA resistance and FQ resistance as well as its potential impact on the treatment outcome, in order to optimize treatment for those patients.

## Supporting information

S1 File Database with all demographic and laboratory testing data. Drug Susceptibility Testing (DST) data are mentioned in association with the technique used.
(XLS)

S2 File Detailed procedure. List of drugs tested and their respective critical concentrations, full standard operating procedure for pncA gene sequencing when starting from culture samples, and list of primers used to sequence targeted genes.
(DOCX)

S3 File List of *pncA* mutations observed in our samples. Individual mutations reported, and their (un)known association with resistance (interpretation).
(XLS)
