# Supplementary material for: Half of rifampicin-resistant Mycobacterium tuberculosis complex isolated from tuberculosis patients in Sub-Saharan Africa have concomitant resistance to pyrazinamide
Source: PLoS One. 2017 Oct 31;12(10):e0187211. doi: 10.1371/journal.pone.0187211 (PMC5663438; doi:10.1371/journal.pone.0187211)
Supplement: S2 File — List of drugs tested and their respective critical concentrations, full standard operating procedure for pncA gene sequencing when starting from culture samples, and list of primers used to sequence targeted genes. (DOCX) [file pone.0187211.s002.docx]

# **File S2**

# **Critical concentration of drugs tested and the Standard Operating Procedure for target gene sequencing**

1. **Critical concentrations for the drugs tested phenotypically**

| **S/N** | **Drugs** | **DST critical concentrations (μg/ml)** | |
| --- | --- | --- | --- |
|  |  | **Löwenstein jensen proportion method** | **MGIT 960** |
| 1 | Rifampicin | 40 | 0.1 |
| 2 | Isoniazid | 0.2 | 1 |
| 3 | Streptomycin | 4 | 1 |
| 4 | Ethambutol | 2 | 5 |
| 5 | Ofloxacin | 2 | NA |
| 6 | Kanamycin | 30 | NA |
| 7 | Capreomycin | 40 | NA |
| 8 | p-Amino salicylic acid | 1 | NA |

1. **Standard Operating Procedure for target gene sequencing**

- **Sample preparation, PCR and gel electrophoresis**
- **Sample preparation**

***General remarks***

Start from culture, so work in a BSL-3 lab or at least laminar flow.

***Culture collection***

- From fresh solid culture, collect a full loop of colonies
- From fresh liquid culture, collect one drop (≈25µl)
- From frozen culture samples, scrap on the side with the corner of a spatula (≈5mm³)

Homogenize in 400µl molecular-grade TE buffer, or distilled water if TE not available

Transfer in sterile Eppendorf with screw cap

Boil 5min (to kill bacteria + lyse membrane to make DNA free)

Sample can go to molecular lab

Store at -20°C or use for PCR

***Annexes***

- TE buffer preparation

Material: 10 mM [Tris](http://en.wikipedia.org/wiki/Tris), bring to pH 8.0 with [HCl](http://en.wikipedia.org/wiki/HCl), and 1 mM [EDTA](http://en.wikipedia.org/wiki/EDTA)

- Measure 10ml of 1M Tris-Cl buffer and 2ml of 0.5M EDTA solution.
- Mix the solutions with distilled H_2_O and make up the volume to 1000ml using a graduated measuring cylinder
- **PCR for pncA or rpoB**

***General remarks***

Work in a DNA-low environment to prepare PCR mix.

Avoid environmental mycobacteria that can false your results.

- Separate pre- and post-PCR (even separate mix preparation, and sample addition)
- Wear gloves
- Work in a laminar flow
- Use sterile tubes and tips with filter
- Pipet only in aliquots, not in stock solutions

***Mix preparation***

*Depends on your reagents (buffer 10x for example, or ready-to-use mix)*

*Here for Qiagen Hotstart master mix, and for 25µl reaction*

Prepare primers working solution at 25pmol/µl (suspension and dilution in TE buffer)

Mix:

| **Reagent** | **1 reaction (µl)** | **5 reactions (µl)** | **10 reactions (µl)** | **20 reactions (µl)** |
| --- | --- | --- | --- | --- |
| Water | 9.9 | 49.5 | 99 | **198** |
| Master mix 2x | 12.5 | 62.5 | 125 | **250** |
| Primer S | 0.8 | 4 | 8 | **16** |
| Primer R | 0.8 | 4 | 8 | **16** |

Number of reactions should include 1 positive + 1 negative control sample, and ̴10% extra.

Distribute 24µl mix in tubes adapted to PCR thermocycler

Add 1µl water in the negative control sample

***Adding samples***

Centrifuge (spin) samples before opening tubes

Add 1µl sample in respective tube, close and mark it

Place all tubes in the thermocycler

Program for *pncA*:

|  | **Denaturation** | **45x cycles** | | | **Final elongation** |
| --- | --- | --- | --- | --- | --- |
|  |  | Denaturation | Annealing | Elongation |  |
| Time | 5min | 1min10 | 1min | 1min30 | 10min |
| Temperature | 95°C | 95°C | 66°C | 72°C | 72°C |

Slow temperature ramp: 1°C/sec; heated lid at 99°C

Program for *rpoB*:

|  | **Denaturation** | **45x cycles** | | | **Final elongation** |
| --- | --- | --- | --- | --- | --- |
|  |  | Denaturation | Annealing | Elongation |  |
| Time | 5min | 1min10 | 1min30 | 3min30 | 10min |
| Temperature | 95°C | 95°C | 66°C | 72°C | 72°C |

- **Agarose gel electrophoresis**

Make an agarose gel at 2% in TAE 0.5%

- Dilute TAE stock (“50x”) 10x with distilled water
- Weigh 1g of agarose and put in Erlenmeyer (adapt proportionally if required volume is different than 100ml)
- Add 100 ml of diluted TAE (or other required volume)
- Heat in microwave until it dissolved
- Cool down until hand warm
- Add 5 µl of ethidium bromide (of 10mg/ml) or 8µl of 1%
- Shake and pour into the gel tray, with a comb adapted to number of samples
- Wait 30minutes for solidifying
- Put it in the electrophoresis tank and fill in with TAE 0.5x buffer up to covering the gel (you may store the gel overnight in buffer, if prepare beforehand)

Load samples (mixed with loading buffer) ; volume depending on well size, recommended 5-10µl

Add molecular ladder 100-1000bp

Migrate 20-30 min at 100V

Check product size

- *pncA*: 1163 bases
- *rpoB*: 2106 bases

Check intensity (should be brighter than molecular weight)

***Annexes***

- TAE buffer 50x

- Resuspend 242 g Tris base in 700 ml distilled water
- Add 7,1 ml acetic acid (100%)
- Add 100 ml 0,5 M EDTA pH 8
- Add water until 1L
- Autoclave and stock at RT° for maximum 1 year

- Ethidium bromide (Intercalating agent!)

Stock solution at 10mg/ml

- Weight 500mg ethidium bromide
- Add 50ml distilled water
- Store at 2-8°C in a brown bottle
- **Preparation of samples to be shipped to Macrogen**

***General remark***

Macrogen proposes sequencing with pre-payment system including PCR product purification and sequencing (Eco-seq labels): create an account, and order through <http://dna.macrogen.com/>

Shipping by DHL or FEDEX is included in the price if you send at least 20 samples at a time

***Sample preparation***

- In pre-PCR room

Calculate the amount of primer required for all your samples: 5µl/sample

Prepare a sequencing primer solution at 5pmol/µl (dilution 5x regarding usual primer working solution, with molecular-grade water, in a sterile tube of 1,5ml, preferably with screw cap)

Identify primer tube by writing “p” or “primer” on the cap and/or side

- In post-PCR room

Paste Eco-seq labels on each tube, with ends pasted on each other (in a sterile tube of 1,5ml, preferably with screw cap)

Add carefully 5µl of DNA in the corresponding labelled tube

Know in which order you add sample vs Eco-seq numbers; avoid using paper sheet to take out of the post-PCR

At the post-PCR limit, place tubes in the Macrogen envelop

Ask DHL or FEDEX; use Macrogen account to prepare air bill (see contact given when ordering)

***Download results from Macrogen***

Results are sent back to the email address linked to your account (or individualised through <http://www.ezseq.com/>)

Results are stored on Macrogen website; the email contains a link to download a ".zip" file that contains all the results (ab1 file and PDF with chromatograms; and txt and phd1 that you don’t use)

Click on the link in the “results” window

In the new internet window, click on “Download click here”

A ZIP file is download (procedure according to your computer), it can take a few minutes

Open the file (you need a ZIP software)

Extract the files (click on “Unzip”), and save then in a folder that you create for sequencing results

- **Using Mega software for sequence analysis**

Free online download, version for Windows, Mac and Linux. Numerous functionalities, easy to handle.

***Install software***

Go on <http://www.megasoftware.net/>

Open exe file, and click on « Run »

Allow the software to make changes on your computer

In the assistant, click each time on « Next » without any change just tick « Create a desktop icon » on the 4^th^ window if you want a desktop icon

Click on « Install » ; the software will open automatically when install finished

***User manual***

***To create a reference file***

Click on « Align », select « Edit/Build alignment  », then select “Create a new alignment”, and “DNA”

Click on « Edit », select « Insert blank sequence »

Paste reference sequence (see end of SOP for pncA and rpoB)

Change name by right click on by-default sequence name (“Sequence 1”), and select « Edit sequence name »

Save file named as the gene reference sequence

***To align DNA sequences***

Click on « Align », select « Open saved alignment session »

Open file with reference sequence (*rpoB* ou *pncA*, or other as applicable)

In « Edit », select « Insert sequence from file »

Open all files to be analyzed (in once, using shift button and click on first then on last from the list)

!!! Select all files (CTRL+ A)

In « Alignment », select « ClustalW »; do not change any option, just click « OK »

Deselect by clicking on any base, to show colors

Check visually if it looks aligned, including with the reference

An asterisk in head line indicates that all nucleotides are identical for this position; for a clearer view of positions with a mutation, in « Display », select « Toggle conserved sites – at 100% »

*To correct nucleotides if required*

Delete sequence parts that are outside the reference region, on the beginning and the end: select columns (click on the asterisk line) and press Delete

If a nucleotide is indeterminate (in gray, other than ACTG) or uncertain (possible mutation/insertion/deletion), in « Sequencer », select « edit sequencer file » and open file from this sample; you get the sequencing chromatogram, with, above, the identified letter for each position, as well as position numbering (by 10; be careful: the 1 corresponds to the first base of this specific sequence, not from the reference!)

Look at the position to be modified, by function « Search » and « Find »; use some nucleotides around the searched one (copy from alignment file by selecting those letters, and CTRL+C ; copy in Find window with CTRL+V, or right click and Copy/Paste respectively)

Note: To move between windows, use icons on the software desk, if your navigator does not show all windows in the current toolbar

Determine the adequate letter according to the chromatogram color (A in green, T in red, G in black, and C in blue); if not visible (peak too low), leave indeterminate letter or consider as not a mutation (same letter than in reference) ; if double color, write as mutation, and write on paper that there is a technical doubt or mixture of sensitive/resistant

In alignment window, select the indeterminate letter, write the right one (just type the letter, it will be inserted), and delete the indeterminate one (press Delete)

If nucleotide too much, just click on the letter and press Delete

If some sequencer files are too bad (no clear peaks for a long part), delete the region from the moment it is no more readable (copy from chromatogram, and Search-Find in the alignment window; then select until the end of the row, and Delete – only for that sequence, not through columns !)

*To save the file*

Save the window, including alignment and corrected letters, via « Data », « Save Session »

Give a new name (not writing on Reference session), including date and your name, and a short description of gene/experiment, for traceability

*To translate to protein*

Be sure your sequence is in Sense direction

- For *rpoB* and *pncA*, sequence is **Reverse**; to get the Sense, select all sequences (CTRL+A), and in « Data », click on « **Reverse** » and do the same with «**Complement**»

Realign if it does not look aligned anymore

If you have some real insertions, creating gaps in the reference sequence, write the inserted letter(s) and the position (number mentioned in the small frame bottom left when you select the first inserted letter – see details further down), then delete individual gaps (not the whole column), that create false numbering in the reference, and wrong translation (select columns, and “Edit”, “Delete gaps”) ; don’t do it before Reverse/Complement !

In « Data », select «Translate/Untranslate»; it asks the genetic code to apply: if this is « Standard », tick the « Use the selected genetic code for rest of the Alignment session », and answer « No »; select « Bacterial plastid »

Like in DNA, an asterisk in head line indicates that all nucleotides are identical for this position; for a clearer view of positions with a mutation, in « Display », select «Toggle conserved sites – at 100%»

Save the window, via « Data », « Save Session », keeping the same name or update with reverse-complement

*Analyze and export to Excel*

Go to « Data », « Phylogenetic analysis »; click on sign « TA » appearing on MEGA desktop to have the « Analyze » window;

Be sure you are in “DNA” when applying “Phylogenetic analysis”, and select “Yes” for protein-coding nucleotide sequence data

Or reopen your file «.mas » via « File » (upper left) or « Data » (at the right of Align), « Open a File/Session », and open in « Analyze »,

NB: Open in « Align » will give you the same interactive window than origin, and you need it only to work further on alignment/translation if not yet finished

The Analyze window (click on TA sign) is open on DNA; to go to protein (or go back to DNA), and export each file, use « Data », « Translate sequence »

You can select the format with points for identical letters (if not automatic, go into « Display », select « Use identical symbol »)

You can highlight the mutations by selecting “Highlight”, “Variable sites”; they appear in yellow (only for the window open at that moment, not for both DNA and protein); be careful: gaps are not highlighted, only mutations are!

If you want to export alignment in a universal/printable format, export to Excel via « Data », « Export Data », select format Excel Workbook (window middle left) ; in format « Writing site numbers », select « for each site »; do it for both DNA and protein (it exports only the data seen on the screen)

NB: in excel, you can fix the first column to always see the sequence names on the screen: select one of the sequence name, then in "View", select "Freeze first column"

Save the excel file with the same name than mas, + precision if DNA or protein

*To position mutations*

In MEGA, Alignment or Analyze window, click on the desired letter (nucleotide or amino acid), you get automatically its position via the mentioned number at the bottom left.

In Analyze window, if you select a position in protein, and then go back to DNA view, you stay at equivalent position (first DNA letter of the selected codon), so you can see the corresponding DNA mutation (and inversely from DNA to protein)

In Excel, numbering is mentioned above each line (vertical view) ; be careful: some computers/Windows/MEGA versions create a mistake in numbering, placing the vertical number two columns more to the left than it should be

Check in the nucleotide sequence for mutations in promotor (138 first nucleotides for *pncA*); report as position number followed by “nucleotide letter in reference/new letter” ; example: G-15A or -15 G/A

Check in the nucleotide sequence for insertions; report as “ins” + “nucleotide letters inserted” ; example: ins45 GG

Check in the nucleotide sequence for deletions; report as “del” + “missing nucleotide letters” or only position, in case of large deletion (with eventually number of nucleotides, if easy to find); example: del45 TCGA, or del52 65bp

Check in the protein sequence for individual mutations; report as “amino acid abbreviation in reference/new abbreviation” (by preference nomenclature in three letters) ; example: Cys14Arg or 14 Cys/Arg

| A | Ala | Alanine |
| --- | --- | --- |
| C | Cys | Cysteine |
| D | Asp | Aspartic acid |
| E | Glu | Glutamic acid |
| F | Phe | Phenylalanine |
| G | Gly | Glycine |
| H | His | Histidine |
| I | Ile | Isoleucine |
| K | Lys | Lysine |
| L | Leu | Leucine |
| M | Met | Methionine |
| N | Asn | Asparagine |
| P | Pro | Proline |
| Q | Gln | Glutamine |
| R | Arg | Arginine |
| S | Ser | Serine |
| T | Thr | Threonine |
| V | Val | Valine |
| W | Trp | Tryptophan |
| Y | Tyr | Tyrosine |

Correct numbering it if the number 1 from the reference sequence is not the first nucleotide in the original gene (and E. coli is the reference nomenclature) [cfr annex for applicable values for *pncA* and *rpoB*]

***To control quality***

Check if your reference sequence is still the same length than the original one, and if start and end regions are identical as control table (cfr annex for applicable values for *pncA* and *rpoB*)

- **Information and quality control for pncA gene**

| Info |  |  |  |
| --- | --- | --- | --- |
| Reference (original DNA) | TCAGGAGCTGCAAACCAACTCGACGCTGGCGGTGCGCATCTCCTCCAGCGCGGCGACGGTGGTATCGGCCGACACACCCGCTGTCAGGTCCACCAGCACCCTGGTGGCCAAGCCATTGCGTACCGCGTCCTCGGCCGTCTGGCGCACACAATGATCGGTGGCAATACCGACCACATCGACCTCATCGACGCCGCGTTGCCGCAGCCAATTCAGCAGTGGCGTGCCGTTCTCGTCGACTCCTTCGAAGCCGCTGTACGCTCCGGTGTAGGCACCCTTGTAGAACACCGCCTCGATTGCCGACGTGTCCAGACTGGGATGGAAGTCCGCGCCGGGAGTACCGCTGACGCAATGCGGTGGCCACGACGAGGAATAGTCCGGTGTGCCGGAGAAGTGGTCACCCGGGTCGATGTGGAAGTCCTTGGTTGCCACGACGTGATGGTAGTCCGCCGCTTCGGCCAGGTAGTCGCTGATGGCGCGGGCCAGCGCGGCGCCACCGGTTACCGCCAGCGAGCCACCCTCGCAGAAGTCGTTCTGCACGTCGACGATGATCAACGCCCGCATACGTCCACCATACGTTCGGGCGACTGCCCGGGCAGTTTGCCTACCGACGCGGCAGCCACAGATATAGGGTCCATGACGCCGCGACGATCGCGAACATGACCAGCTGAGCGGCGGCCACCCAACCGGCGGGATAGATCA | | |
| Primer PCR forward | pncA-S:GGCCCGATGAAGGTGTCGTAGAAGC | | |
| Primer PCR reverse | pncA-R:CGACCTGGAAAGGCAACCCGAGAG | | |
| Primer sequencing (sense) | pncA-S:GGCCCGATGAAGGTGTCGTAGAAGC (idem PCR) | | |
| Sequences should reverse + complement | yes | (gene is biologically read anti-sense) | |
| Correction position DNA (promotor) | -139 | promotor = nucleotides 1 to 138 | |
| Correction position DNA (gene) | -138 | gene = starting at nucleotide 139 | |
| Correction position protein | -46 | gene = starting at amino acid 47 | |
|  |  |  |  |
| Quality control |  |  |  |
| Reference length should be | 699 |  |  |
| Reference DNA should start with | TGATCTATCC |  |  |
| Reference DNA should finish with | CAGCTCCTGA |  |  |
| Reference protein should start with | *SIPPVGWPP |  |  |
| Reference protein should finish with | TASVELVCSS* |  |  |
|  |  |  |  |
| Troubleshooting |  |  |  |
| If reference start with | TCAGGAGCTG | you did not reverse-complement | |
|  | ACTAGATAGG | you did reverse but not complement | |
|  | AGTCCTCGAC | you did complement but not reverse | |

- **Information and quality control for rpoB gene**

| Info |  |  |
| --- | --- | --- |
| Reference (original DNA) | GCTGCATGTTTGCCCCCATGAGGGCACGGTTGGCGTCGTCGTGCTCCAGGAAGGGAATCATCGCGGTGGCCACCGACACCATCTGGCGGGGCGAGACGTCCATGTAGTCCACCTCAGACGAGGGCACGTACTCCACCTCGCCCGCCTTGCGGCGGACCAGCACGCGCGGCTCGACGAAGCGACCGTCCGCATCGATCGGCGAATTGGCCTGTGCCACCACGTGGCGGTCCTCCTCGTCGGCGGTCAGGTACACGATCTCGTCGCTAACCACGCCGTCGACCACCTTGCGGTACGGCGTTTCGATGAACCCGAACGGGTTGACCCGCGCGTACACCGACAGCGAGCCGATCAGACCGATGTTGGGCCCCTCAGGGGTTTCGATCGGGCACATCCGGCCGTAGTGCGACGGGTGCACGTCGCGGACCTCCAGCCCGGCACGCTCACGTGACAGACCGCCGGGCCCCAGCGCCGACAGTCGGCGCTTGTGGGTCAACCCCGACAGCGGGTTGTTCTGGTCCATGAATTGGCTCAGCTGGCTGGTGCCGAAGAACTCCTTGATCGCGGCGACCACCGGCCGGATGTTGATCAACGTCTGCGGTGTGATCGCCTCCACGTCCTGGGTGGTCATCCGCTCCCGGACCACCCGCTCCATCCGCGACATGCCGACCCGGATCTGGTTTTGGATCAGCTCGCCGACCGTACGCAGGCGGCGGTTGCCGAAGTGGTCGATGTCGTCGGTTTCCACCGGCACCTCGACGCCGCCCGGAACGGTCATCGTGGTCTGACCCTCGTGCAAGCGGACCAGATATTCGATGGTGGCCACGACGTCTTCTTCGGTCAGCGTCGACGACGTGATGGGCTCGCCGACATGCAGCCCGAGCTTCTTGTTGACCTTATAGCGACCGACGCGGGCCAGGTCGTAGCGCTTCTCCTTGAAGAACAAGTTTTCCAACAGCGTCTGCGCTGACTCTTTGGTCGGGGGCTCGCCCGGACGCAGCTT | |
| Primer PCR forward | rpoBgen-SAnew :  5’-GCAAAACAGCCGCTAGTCCTAGTCCGA-3’ | |
| Primer PCR reverse | rpoBgen-RA : 5’-GCGCCATCTCGCCGTCGTCAGTACAG-3’ | |
| Primer sequencing (reverse) | rpoBgene-R1: 5’-CAGCGGGGCCTCGCTAC-3’ (sequencing of the reverse strand, the best one) | |
| Sequences should reverse + complement | yes | (sequencing is reverse) |
| Correction position DNA | +1062 |  |
| Correction position protein | +354 |  |
|  |  |  |
| Quality control |  |  |
| Reference length should be | 1000 |  |
| Reference DNA should start with | AAGCTGCGTC |  |
| Reference DNA should finish with | AACATGCAGC |  |
| Reference protein should start with | KLRPGEPPTK |  |
| Reference protein should finish with | NRALMGANMQ |  |
|  | | |
| Troubleshooting |  |  |
| If reference start with | GCTGCATGTT | you did not reverse-complement |
|  | TTCGACGCAG | you did reverse but not complement |
|  | CGACGTACAA | you did complement but not reverse |

- **Primers used for PCR and sequencing of TB genes related to drugs resistance.**

| Gene | Primer sequence |
| --- | --- |
| *pncA* PCR forward* | GGCCCGATGAAGGTGTCGTAGAAGC |
| *pncA* PCR Reverse* | CGACCTGGAAAGGCAACCCGAGAG |
| *pncA* sequencing | GGCCCGATGAAGGTGTCGTAGAAGC |
| *rpoB* PCR forward | GCAAAACAGCCGCTAGTCCTAGTCCGA |
| *rpoB* PCR reverse | GCGCCATCTCGCCGTCGTCAGTACAG |
| *rpoB* PCR sequencing | CAGCGGGGCCTCGCTAC |
| *embB* PCR forward | AATTGCCCAGCTCCTCCTCAG |
| *embB* PCR reverse | GCTCGATCAGCACATAGGTGAC |
| *embB* sequencing | AATTGCCCAGCTCCTCCTCAG |
| *gyrAB* PCR forward | TAAGAGCGCCACCGACATCGGTGGATTG |
| *gyrAB* PCR reverse | GATGAAATCGACTGTCTCCTCGTCGATTTCCC |
| *gyrAB* sequencing forward | TAAGAGCGCCACCGACATCGGTGGATTG |
| *gyrAB* sequencing reverse | GTCGATTTCCCTCAGCATCTCCATC |
| *rrs* PCR forward | AAGTACCCCGCCTGGGGAGTACGG |
| *rrs* PCR reverse | GGTGGGACAACACCTGGAACAAGTC |
| *rrs* sequencing | AAGTACCCCGCCTGGGGAGTACGG |
| *eis* PCR forward | TCGACCGGGTTTGCCAGCTTGTC |
| *eis* PCR reverse | CCAGGCACCGTCAACCGCAGA |
| *eis* sequencing | CCAGGCACCGTCAACCGCAGA |
| *tly* PCR forward | CGACGTCGGTGGTGGTGCGGTA |
| *tly* PCR reverse | GTCCGGTCTTCCACCCGGTAATCCT |
| *tly* sequencing | GCATCGCACGTCGTCTTTCCGA |

* *pncA* primers used in Milan: ATGTTCCGGCGGTGATATG and GGGAATGAACACCGTCACA
